# Supplementary material for: SyNDI: synchronous network data integration framework
Source: BMC Bioinformatics. 2018 Nov 6;19:403. doi: 10.1186/s12859-018-2426-5 (PMC6219086; doi:10.1186/s12859-018-2426-5)
Supplement: Supplementary file 5 — Files of the example presented in Additional file 4. This additional file is a zip package that contains all files used in Additional file 4. Detailed descriptions of each of these files are presented in a README file included in this zip package. (GZ 30411 kb) [file 12859_2018_2426_MOESM5_ESM.gz › SyNDITools/Meme2Fimo/meme_4.9.0_4.tar.gz!/meme_4.9.0/website/html/intro.html]

MEME Suite - Introduction


# MEME Suite

The MEME Suite web application requires the use of JavaScript
  
Javascript doesn't seem to be available on your browser.


# The MEME Suite allows you to:

- discover motifs using
  MEME,
  DREME (DNA only)
  or
  GLAM2
  on groups of related DNA or protein sequences,
- search sequence databases with motifs using
  MAST,
  FIMO,
  MCAST or
  GLAM2SCAN,
- compare a motif to
  all motifs in a database of motifs,
- associate motifs with Gene
  Ontology terms via their putative target genes, and
- analyse motif enrichment using
  SpaMo or
  CentriMo.

# To submit a query, click on one of the logos below or select "Submit A Job" from the menu at the left.

---

|  |  |  |  |  |
| --- | --- | --- | --- | --- |
|  |  |  |  |  |
|  |  |  |  |  |
|  |  |  |  |  |
|  |  |  |  |  |

---

### Maintenance and development of the MEME Suite is funded by the National Center for Research Resources grant NIH/NCRR R01 RR021692. The MEME Suite web server is funded by the National Biomedical Computation Resource.

---

**Developed and maintained by:**

|  |  |  |  |
| --- | --- | --- | --- |
|  |  |  |  |

|  |  |  |
| --- | --- | --- |
|  |  |  |
